# Genome analysis and antagonistic activity of *Streptomyces* sp. strain J36 against *Phytophthora cactorum*

**DOI:** 10.3389/fmicb.2026.1922030

**Published:** 2026-08-21

**Authors:** Shuang Ma, Lisha Jiang, Die Yang, Gao Xiong, Mengmeng Cheng, Zhongjian Chen, Teng Wang, Yong Wang

**Affiliations:** Wenshan Sanqi Institute of Science and Technology, College of Notoginseng Medicine and Pharmacy, Wenshan University, Wenshan, China

**Keywords:** antagonistic mechanism, biological control, *Panax notoginseng*, *Phytophthora cactorum*, *Streptomyces*

## Abstract

The Phytophthora blight of *Panax notoginseng*, caused by *Phytophthora cactorum*, is a devastating oomycete disease. Biocontrol strategies hold immense potential for inhibiting the spread of *P. cactorum*. We isolated 72 actinobacteria from soil and screened their antagonistic activity against *P. cactorum*. Both strain J36 and its cell-free filtrate exhibited strong antagonistic activity against *P. cactorum* and were therefore selected. Based on the 16S rRNA gene phylogenetic tree, strain J36 formed a well supported subclade with *Streptomyces zaomyceticus* NRRL B-2038 (bootstrap value 100%). However, because 16S rRNA sequences often lack sufficient resolution for species-level discrimination, we performed multilocus sequence analysis (MLSA) using three housekeeping genes (rpoB, recA, and atpD). The MLSA results consistently placed strain J36 within the same cluster as *S. zaomyceticus* NRRL B-2038, with a bootstrap support of 99%, indicating a close phylogenetic relationship. To further clarify the taxonomic status, we calculated the average nucleotide identity (ANI) and digital DNA–DNA hybridization (dDDH) values between strain J36 and the type strain of *S. zaomyceticus* NBC-00415^T^. The ANI value was 90.91% and the dDDH value was 39.30%, both well below the accepted thresholds for species demarcation (ANI < 95%, dDDH < 70%). These genomic indices therefore strongly support that strain J36 represents a novel species within the genus *Streptomyces*. Through whole-genome sequencing and CAZymes analysis, a total of 98 carbohydrate-active enzymes (CAZymes) were detected, including 2 cellulase and 2 β-1,3-glucanases. The cell-free filtrate, which exhibited strong antagonistic activity against *P. cactorum*, also showed high activities of cellulase and β-1,3-glucanase, suggesting that these enzymes may be involved in its anti-oomycete activity. These findings suggest that J36 has potential as a biocontrol candidate, although further *in vivo* evaluation is needed to confirm its efficacy against *Phytophthora* blight of *P. notoginseng*.

## Introduction

*Panax notoginseng* (Burk.) F. H. Chen is an important medicinal crop in the Wenshan region of China. However, the *P. notoginseng* industry is severely threatened by root rot caused by *Phytophthora cactorum*, a soil-borne oomycete belonging to the order Peronosporales and the genus *Phytophthora* (Guo et al., 2009). Although root rot of *P. notoginseng* was first reported in the early 2000s, the efficacy of various biological control agents against *P. notoginseng* root rot has been demonstrated (Liu H. et al., 2025; Liu Y. P. et al., 2025; Wang et al., 2023; Li et al., 2022). Nevertheless, the complex mechanisms governing the interactions among antagonistic microorganisms, the pathogen, and *P. notoginseng* remain poorly understood. Therefore, further research into the use of antagonistic microorganisms to control *P. cactorum* is a promising strategy due to its environmental friendliness and high efficiency.

Actinobacteria are widely distributed in soil and natural environments and are considered ideal candidates for biocontrol agents (Bhatti et al., 2017). Their colonization of the plant rhizosphere and potential for controlling soil-borne diseases have received extensive attention. As the largest genus within the phylum Actinomycetota, Streptomyces exhibits a Gram-positive, aerobic, filamentous morphology, with a genomic G + C content of approximately 70% (Qi et al., 2024). Current identification of *Streptomyces* typically combines phenotypic and genotypic characteristics, but the resolution of 16S rRNA sequences is often insufficient at the species level. Multilocus sequence analysis (MLSA) provides a more effective means for species-level identification of *Streptomyces*, especially when distinguishing among multiple candidate species (Hu et al., 2022). The cell walls of oomycetes are mainly composed of cellulose and β-1,3/β-1,6-glucans, and the cellulases and glucanases within the CAZyme family can hydrolyze these components, thereby effectively degrading oomycete cell walls (Liang et al., 2020). *Streptomyces* and other actinobacteria, with their rich CAZyme-secreting capabilities, are ideal candidates for controlling oomycete diseases. However, in the practical production of *P. notoginseng*, there is still a lack of candidate *Streptomyces* strains with high adaptability and strong antimicrobial activity, and due to differences in physiological characteristics among strains, their specific mechanisms of antagonizing pathogens remain unclear. Therefore, isolating and screening *Streptomyces* strains with strong antagonistic activity and good environmental adaptability from specific ecological niches is of great significance for developing efficient and stable biocontrol agents, especially against soil-borne diseases such as Phytophthora blight of *P. notoginseng*.

In this study, 72 actinobacteria were isolated from a *P. notoginseng* planting base in Wenshan City, Wenshan Zhuang and Miao Autonomous Prefecture, China, and screened for their antagonistic activity against *P. cactorum*. Strain J36 and its cell-free filtrate exhibited strong antagonistic activity against *P. cactorum*, leading to its selection for further study. Subsequently, the taxonomic status of strain J36 was determined based on its phenotypic and genotypic characteristics. To explore the anti-oomycete mechanism of strain J36, CAZymes in its genome were predicted, and the anti-oomycete activity of its cell-free filtrate was assayed. In addition, the activities of cellulase and β-1,3-glucanase in the cell-free filtrate of strain J36 were measured.

## Materials and methods

### Isolation of actinobacteria

Soil samples were collected from healthy plots of a *P. notoginseng* planting base in Wenshan City, Yunnan Province, China. Actinobacteria were isolated using the dilution plating method on Gause’s No.1 medium (containing 10 g soluble starch, 0.5 g NaCl, 0.5 g K_2_HPO_4_, 1 g KNO_3_, 0.5 g MgSO_4_, 0.01 g FeSO_4_, 17 g agar, and 1 L distilled water, pH 7.2–7.4). Cycloheximide (50 mg/L) and nalidixic acid (50 mg/L) were added to inhibit the growth of contaminating fungi and bacteria, respectively. The soil samples were sieved through a 0.2 mm mesh, and 10 g of each sample was suspended in 100 mL of sterile water. After incubation at room temperature for 30 min, the soil homogenate was serially diluted with sterile water to 10^−2^, 10^−3^, 10^−4^, and 10^−5^, and the dilutions were spread onto Gause’s No.1 agar plates. The plates were incubated at 28 °C for 5 d, and colonies with different morphologies (shape and color) were picked and further purified on Gause’s No. 1 medium. All purified isolates were stored on Gause’s No.1 solid medium at 4 °C and also in 30% glycerol suspensions in cryotubes at −80 °C.

### Screening of antagonistic actinobacteria against *Phytophthora cactorum*

The antagonistic activity of actinobacteria against *P. cactorum* was screened using the dual culture assay. The isolates were streaked onto the center of potato dextrose agar (PDA) plates, and a mycelial plug of *P. cactorum* was then placed at the 1/4 position of the PDA plate. Plates inoculated with only the *P. cactorum* plug served as the control, and the experiment was performed in triplicate. After incubation at 25 °C for 7 days, the radius of *P. cactorum* was measured, and the antagonistic activity of actinobacteria was calculated according to the following formula: MI = [(C-T)/C] × 100%, where C and T represent the growth radius of the control and treatment groups, respectively.

### Assay of anti-oomycete activity of cell-free filtrate of antagonistic strain

To assess the anti-oomycete activity of the cell-free filtrate of antagonistic strain, the strains were inoculated into 100 mL of liquid Gause’s No.1 medium and incubated at 28 °C with shaking at 180 r/min for 5 days. The supernatant was then filtered through a 0.22 μm sterile filter. A double-layer plate method was used to assess the antagonistic activity of the cell-free filtrate (filtrate concentration 1:1). A mycelial plug of *P. cactorum* (6 mm in diameter) was placed on the double-layer plate. Among the tested strains, strain J36 and its cell-free filtrate exhibited strong antagonistic activity against *P. cactorum* and were selected for further study.

### Cultural and morphological characteristics of strain J36

Strain J36 was inoculated on ISP2, ISP3 (oatmeal agar), ISP4 (inorganic salts-starch agar), ISP5 (glycerol-asparagine agar), ISP6 (peptone-yeast extract iron agar), ISP7 (tyrosine agar), PDA, and Gause’s No.1 agar, and incubated at 28 °C for 5 days (Shirling and Gottlieb, 1966). The cultural characteristics, such as the growth of aerial mycelium and substrate mycelium, as well as the production of soluble pigments, were observed. The Gram staining of strain J36 was performed according to the standard protocol. After growth on Gause’s No.1 agar for 5 days, the spore chains and spore morphology of strain J36 were observed using scanning electron microscopy (Qi et al., 2021).

### Physiological and biochemical characteristics of strain J36

The physiological and biochemical characteristics of strain J36 were determined, including nitrate reduction, gelatin liquefaction, oxidase activity, starch hydrolysis, hydrogen sulfide production, indole production, urease production, siderophore production, antibiotic susceptibility test, and the ability to utilize carbon sources such as glucose, mannitol, arabinose, and maltose (Qi et al., 2021).

### Phylogenetic analysis of strain J36

The strain J36 was inoculated in Gause’s No. 1 liquid medium and incubated at 28 °C with shaking at 180 r/min for 5 d. Genomic DNA was extracted using a Bacterial Genomic DNA Rapid Extraction Kit (Sangon Biotech (Shanghai) Co., Ltd.). For phylogenetic identification of actinobacterial isolates, the 16S rRNA gene was amplified with custom-modified oligonucleotide primers. The core binding regions of the primer set correspond to the conserved sites of canonical primers 27F and 1492R (Weisburg et al., 1991), while extra GC-rich nucleotides were appended to the 5′ ends to reduce non-specific amplification. The primer sequences were as follows: forward 5′-GCTCAGGACGAACGCTGGCGG-3′, reverse 5′-CCTTCCGGTACGGCTACCT TGTTACG-3′, with an annealing temperature of 60 °C for amplifying the 16S rRNA sequence. After sequencing, the 16S rRNA sequences were compared against the EzBioCloud and NCBI databases. Phylogenetic trees were constructed using the maximum likelihood method in MEGA 11.0. Multilocus sequence analysis (MLSA) was performed based on three housekeeping genes, including rpoB, recA, and atpD (Rong and Huang, 2010). The homologous sequences of these three housekeeping genes from other species were downloaded from GenBank, and phylogenetic trees were constructed using the method described above.

### The overall genome-related indexes

To further identify strain J36, we calculated its overall genome-related indexes (OGRIs), such as average nucleotide identity (ANI) and digital DNA–DNA hybridization (dDDH), against related type strains using OrthoANIu (via EZBioCloud, https://www.ezbiocloud.net/tools/ani) and GGDC version 3.0 (Meier-Kolthoff et al., 2022), respectively.

### Genome sequencing and feature analysis of strain J36

The strain J36 was cultivated in ISP4 liquid medium at 28 °C for 3 days. Following centrifugation, the mycelial biomass was harvested and immediately frozen in liquid nitrogen. Whole-genome sequencing was performed on an Illumina Hiseq combined with PacBio platform (Majorbio Bio-Pharm Technology Co., Ltd., Shanghai, China). Raw sequencing data were trimmed and quality-filtered using Trimmomatic (v0.36) (Bolger et al., 2014), and the resulting high-quality reads were assembled into contigs with Unicycler (v0.4.8) (Wick et al., 2017). Putative protein-coding genes were identified using three separate gene-prediction tools: Glimmer (v3.02) (Delcher et al., 2007), Prodigal (v2.6.3) (Hyatt et al., 2010), and GeneMarkS (v4.3) (Besemer and Borodovsky, 2005). Genome annotation was subsequently conducted through NCBI’s Prokaryotic Genome Annotation Pipeline (Tatusova et al., 2016). The prediction of carbohydrate-active enzymes (CAZymes) was initially made via the online CAZy v6 web server (Cantarel et al., 2009). Gene clusters involved in secondary metabolite synthesis were identified using antiSMASH 8.0.4 (Naughton et al., 2017).

### Cellulase and β-1,3-glucanase activity analysis of cell-free filtrate of strain J36

The activities of CAZymes, including cellulase and glucanase, were measured as follows. Three 6-mm mycelial plugs of strain J36 were inoculated into 100 mL of liquid Gause’s No. 1 medium and incubated at 28 °C with shaking at 180 r/min for 5 days. The culture supernatant was collected by centrifugation at 10,000 r/min for 10 min, and then filtered through a 0.22 μm membrane filter to obtain the cell-free filtrate. The activities of cellulase and β-1,3-glucanase were determined using a cellulase activity assay kit and a microbial β-1,3-glucanase ELISA kit (Jiangsu Aidisheng Biotechnology Co., Ltd.), respectively. One unit of cellulase activity was defined as the amount of enzyme required to produce 1 μg of glucose from carboxymethyl cellulose per milliliter of sample solution per minute at 37 °C. One unit of β-1,3-glucanase activity was expressed as the amount of enzyme required to produce 1 μg of glucose from laminarin per milliliter of sample solution per minute at 37 °C. All experiments were performed in triplicate.

### Effect of temperature on the anti-oomycete activity of the fermentation broth

To evaluate the thermal stability of the bioactive metabolites produced by strain J36, the cell-free fermentation broth (prepared as described above) was subjected to heat treatment at 60 °C and 100 °C for 30 min, and at 121 °C for 20 min (autoclaving). After heat treatment, the samples were cooled to room temperature immediately. The residual anti-oomycete activity of the heat-treated fermentation broth was determined using the Oxford cup assay. Briefly, a 6 mm mycelial plug of *P. cactorum* was placed at the center of a PDA plate. Sterile Oxford cups (6 mm in inner diameter) were placed at a distance of 2.5 cm from the mycelial plug. Each cup was filled with 100 μL of the heat-treated fermentation broth, while untreated fermentation broth and sterile culture medium served as the positive and negative controls, respectively. The plates were incubated at 25 °C for 7 days. The diameters of the inhibition zones were measured, and all experiments were performed in triplicate.

## Results

### Isolation and screening of antagonistic actinobacteria against *Phytophthora cactorum*

**Figure 1:**
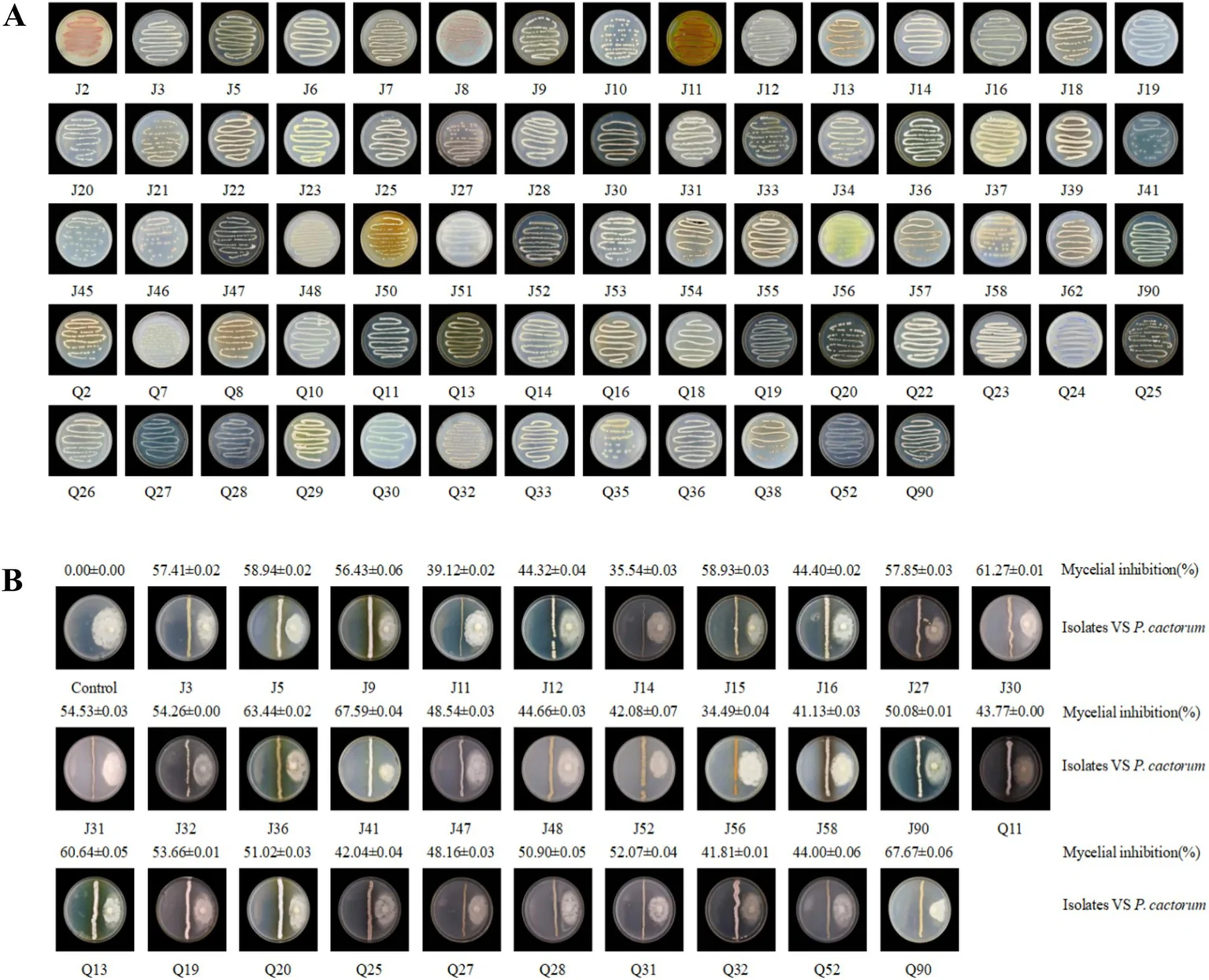
Isolation of actinobacteria and screening of their antagonistic activity against *P. cactorum*. **(A)** Isolation of 72 actinobacterial strains from rhizosphere and root-zone soil samples of *P. notoginseng*. **(B)** Antagonistic activity of 31 actinobacterial strains against *P. cactorum*.

A total of 72 actinobacterial strains were isolated from healthy soil samples collected from a *P. notoginseng* planting base in Wenshan that was infected with *Phytophthora cactorum* (Figure 1A). The antagonistic activity of these isolates against *P. cactorum* was evaluated using the dual-culture assay. Among them, 31 strains showed antagonistic activity against *P. cactorum* (Figure 1B). Among these, strain Q90 exhibited the highest mycelial inhibition rate (67.67 ± 0.06)%, followed by strain J41 (67.59 ± 0.04)%, strain J36 (63.44 ± 0.02)%, strain J30 (61.27 ± 0.01)%, and strain Q13 (60.64 ± 0.05)%.

### Analysis of antagonistic activity of cell-free filtrates from antagonistic strains

**Figure 2:**
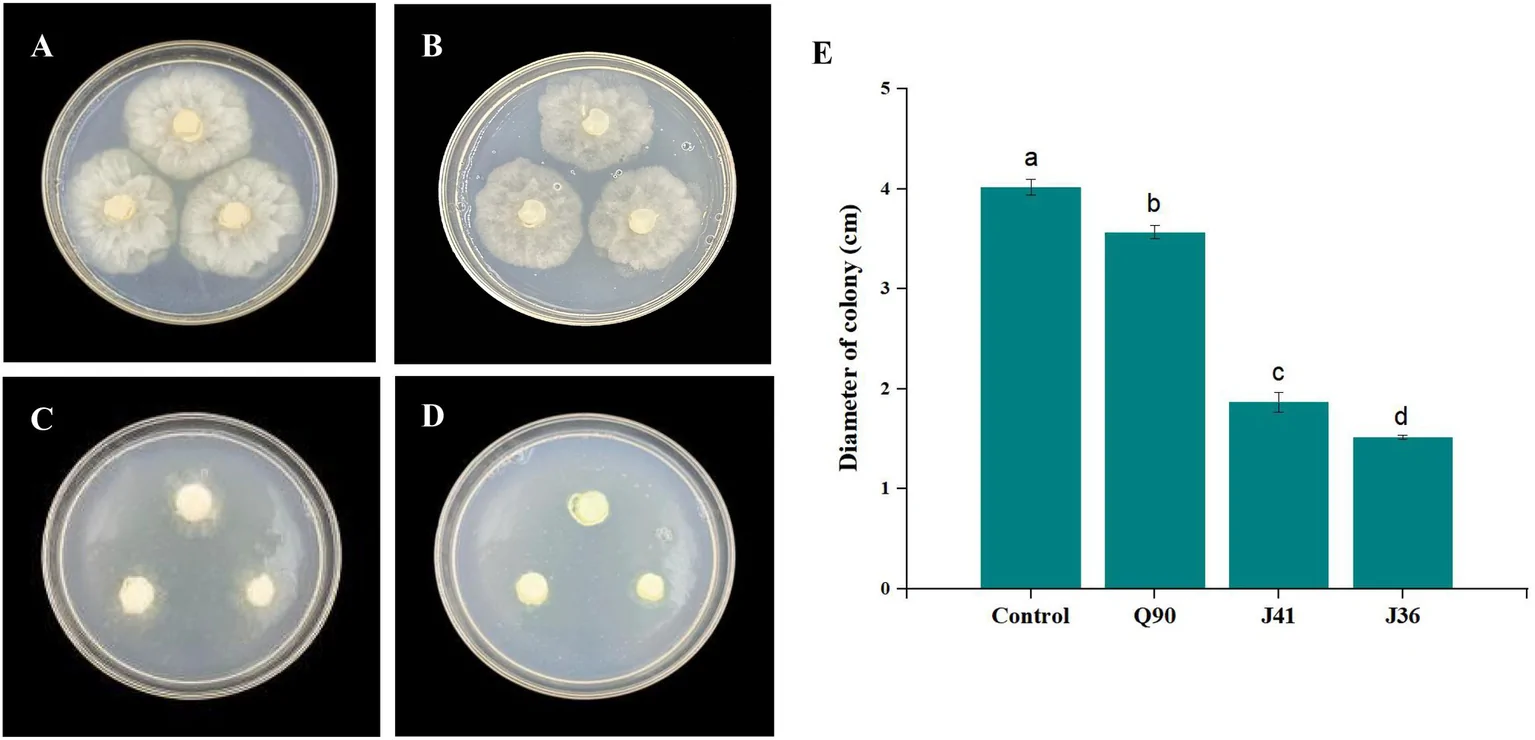
Determination of the anti-oomycete activity of the cell-free filtrate of the top three antagonistic strains. **(A)** Untreated control; **(B)** Treated with 50% (v/v) filtrate of strain Q90; **(C)** Treated with 50% (v/v) filtrate of strain J41; **(D)** Treated with 50% (v/v) filtrate of strain J36; **(E)** Inhibition zone diameters (*n* = 3). Different lowercase letters (a-d) above the bars indicate significant differences (*p* < 0.01) according to one-way ANOVA followed by Duncan’s multiple range test.

Among the 31 antagonistic strains obtained from the primary dual-culture screening, the top three strains (Q90, J41, and J36) were selected for further evaluation of their cell-free filtrate activity using the double-layer plate confrontation assay. At a concentration of 50% (v/v), the sterile filtrate of J36 exhibited the strongest inhibitory activity against the mycelial growth of *P. cactorum*, significantly reducing the colony diameter compared with the other two strains and the control (Figures 2A–E). Therefore, strain J36 was selected for subsequent experiments.

### Cultural morphological characteristics of strain J36

**Figure 3:**
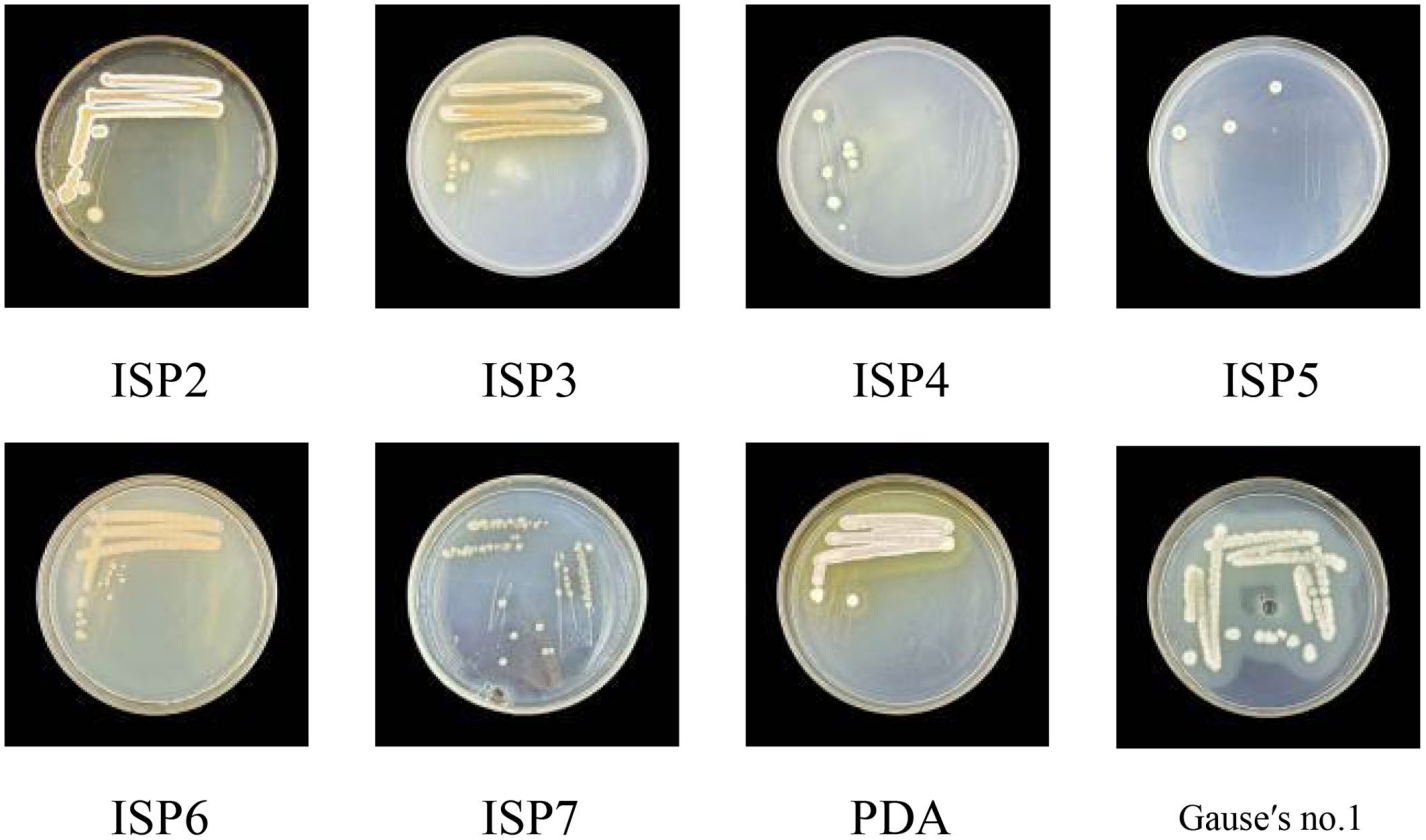
Growth of strain J36 on different differential media.

**Figure 4:**
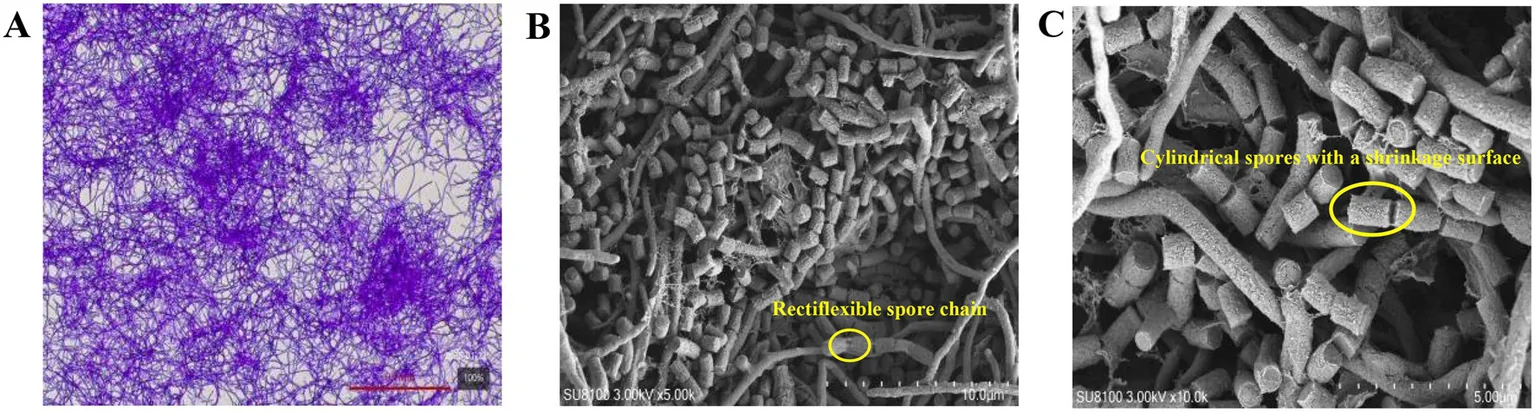
Microstructural characterization of strain J36. **(A)** Gram-stained mycelia observed under light microscopy (scale bar = 100 μm). **(B,C)** Ultrastructural features observed under electron microscopy (scale bars = 10 μm and 5 μm, respectively).

Strain J36 exhibited good growth on ISP2, ISP3, ISP6, PDA, and Gause’s No.1 agar, with moderate growth on ISP7, ISP4, and ISP5. The colony colors on various media ranged from yellow, sandy yellow, silver gray, to milky white (Figure 3). Soluble pigments of light yellow and milky white were produced by strain J36 only on ISP3, PDA, and Gause’s No.1 agar (Table 1). Strain J36 is an aerobic Gram-positive bacterium (Figure 4A). Scanning electron microscopy (SEM) observation of strain J36 on Gause’s No.1 agar revealed the presence of rectiflexible spore chains (Figure 4B) and cylindrical spores with a wrinkled surface (Figure 4C).

**Table 1:** Cultural characteristics of strain J36 on eight different media.

Culture medium	Aerial hyphae	Vegetative mycelium	Soluble pigment	Colony characteristics	Growth conditions
ISP2	White	Yellow	None	Wrinkled, hard	+++
ISP3	Milky white	Yellow	Pale yellow	Loose, wrinkle-free	+++
ISP4	White	Milky white	None	Loose, wrinkle-free	+
ISP5	White	Milky white	None	Loose, wrinkle-free	+
ISP6	Yellow	Yellow	None	Wrinkled, hard	+++
ISP7	Sand yellow	Sand yellow	None	Compact, wrinkle-free	++
PDA	White	Silvery gray	Pale yellow	Compact, wrinkle-free	+++
Gause’s no.1	White	Milky white	Milky white	Loose, wrinkle-free	+++

“+++” represented that the strain grew well; “++” represented the general growth of the strain; “+” represented that the strain could grow.

### Physiological and biochemical features of strain J36

**Figure 5:**
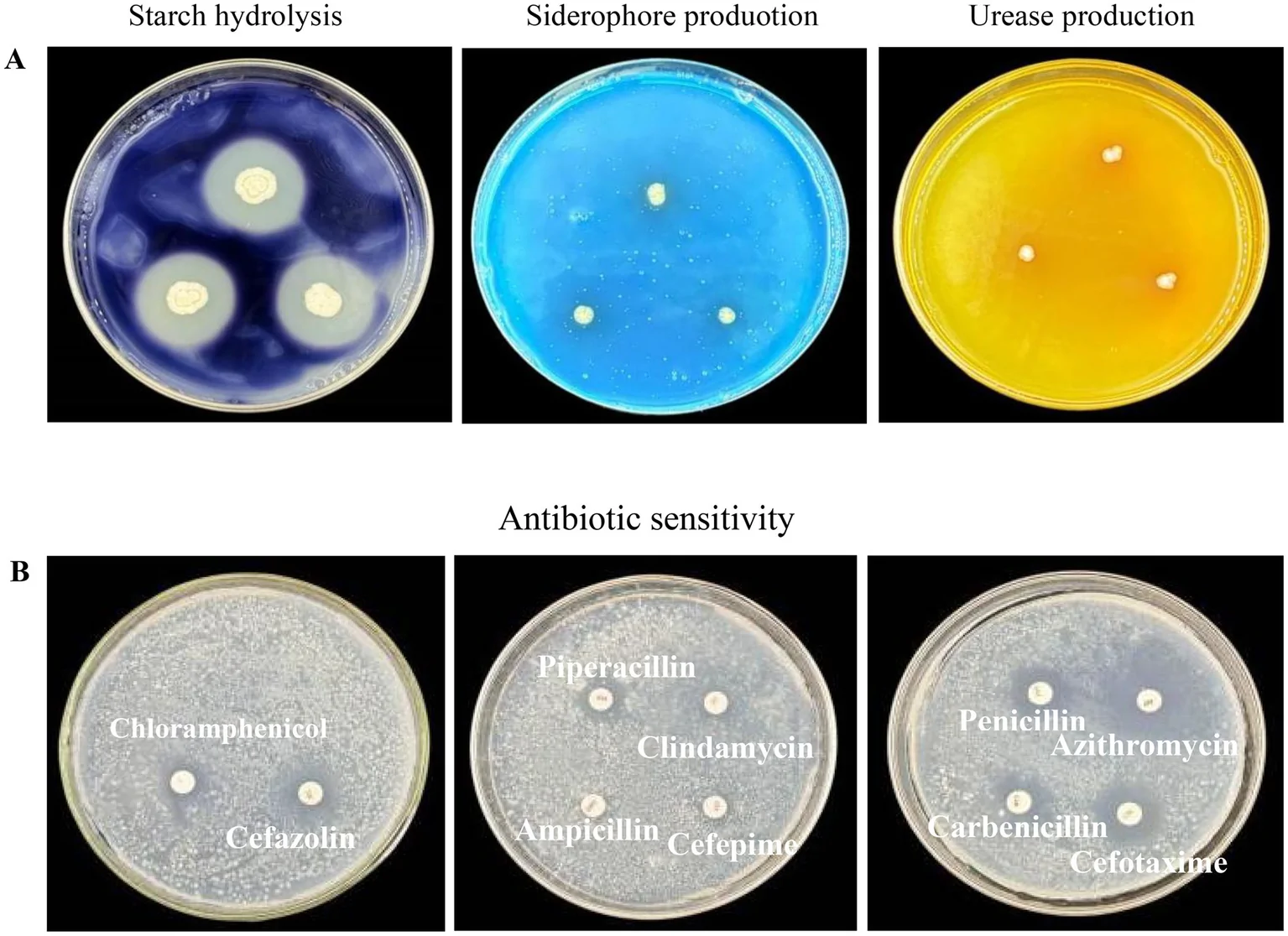
Physiological and chemotaxonomic features of strain J36. **(A)** Production of amylase, siderophores, and urease by strain J36. **(B)** Antibiotic susceptibility profile: The strain is resistant to cefepime, ampicillin, and clindamycin, and susceptible to penicillin, cefazolin, chloramphenicol, piperacillin, cefotaxime, carbenicillin, and azithromycin.

Strain J36 could degrade starch, urea (Figure 5A), gelatin, and nitrate (Table 2), while also producing siderophores and hydrogen sulfide. The strain exhibited resistance to three antibiotics, namely cefepime, ampicillin, and clindamycin, but was sensitive to seven antibiotics, namely penicillin, cephalexin, chloramphenicol, piperacillin, cefotaxime, carbenicillin, and azithromycin (Figure 5B). Furthermore, the strain was able to grow using glucose, mannitol, arabinose, and maltose as carbon sources, but could not produce indole (Table 2).

**Table 2:** Physiological and biochemical characteristics of strain J36.

Characteristic	Test result	Characteristic	Test result
Nitrate reduction	+	Glucose utilization	+
Gelatin liquefaction	+	Mannitol utilization	+
Oxidase activity	−	Arabinose utilization	+
Starch hydrolysis	+	Maltose utilization	+
H_2_S production	+	Indole production	−

### Genome sequencing and characterization of strain J36

**Figure 6:**
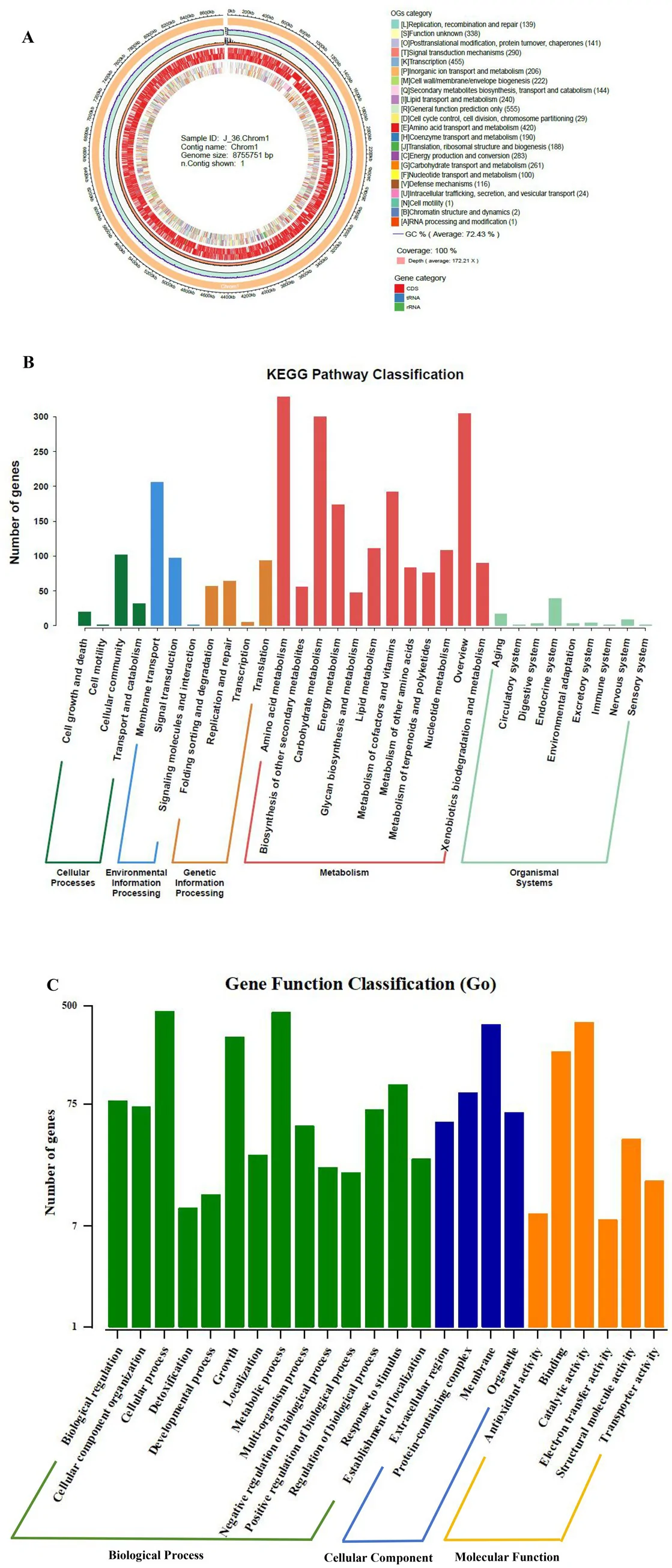
Whole-genome overview, GO functional annotation, and KEGG pathway classification analysis of strain J36. **(A)** Circos circular map of the strain J36 genome. From outside to inside: The outermost ring shows the genomic coordinate scale (bp); the colored bands represent genes assigned to COG functional categories; the red, blue, and green bands indicate CDS, tRNA, and rRNA genes, respectively; the inner curve displays the GC content distribution across the genome. The embedded legend lists each COG functional category along with the corresponding gene count. **(B)** Bar chart summarizing the level-1 KEGG pathway classification of coding genes. **(C)** Bar chart showing the GO functional annotation classification of coding genes.

The genome of strain J36 exhibits typical actinobacterial characteristics. Its genome was sequenced and assembled into a single linear chromosome of 8,755,751 bp, with a GC content of 72.43% (Figure 6A and Table 3). It comprises a total of 7,884 genes, including 7,779 protein-coding genes (CDSs) (Table 3). Genome annotation revealed the presence of 83 tRNA genes, 21 rRNA genes, and 1 sRNA gene, along with 3,327 predicted repeat sequences. No CRISPR-Cas systems or insertion sequences were detected. Based on the COG, KEGG, and GO databases, 4,398, 1,539, and 740 genes were successfully annotated, respectively (Figures 6B,C).

**Table 3:** Genome features of strain J36.

Feature	Chromosome characteristics
Genome size (bp)	8,755,751
Chromosome No.	1
GC Content (%)	72.43
Depth	174
Protein-coding genes No.	7,779
Gene total length (bp)	7,899,615
Gene average length (bp)	1007.07
Gene density	0.97
tRNA genes No.	83
Type of tRNAs No.	22
rRNA genes No.	21
16S rRNA No.	7
23S rRNA No.	7
5S rRNA No.	7
sRNA No.	1
Repeat No.	3,372
CRISPR-Cas No.	0
Insert sequences No.	0
Genes assigned to COG No.	4,398
Genes assigned to KEGG No.	1,539
Genes assigned to GO No.	740

### Phylogenetic analysis of strain J36

**Figure 7:**
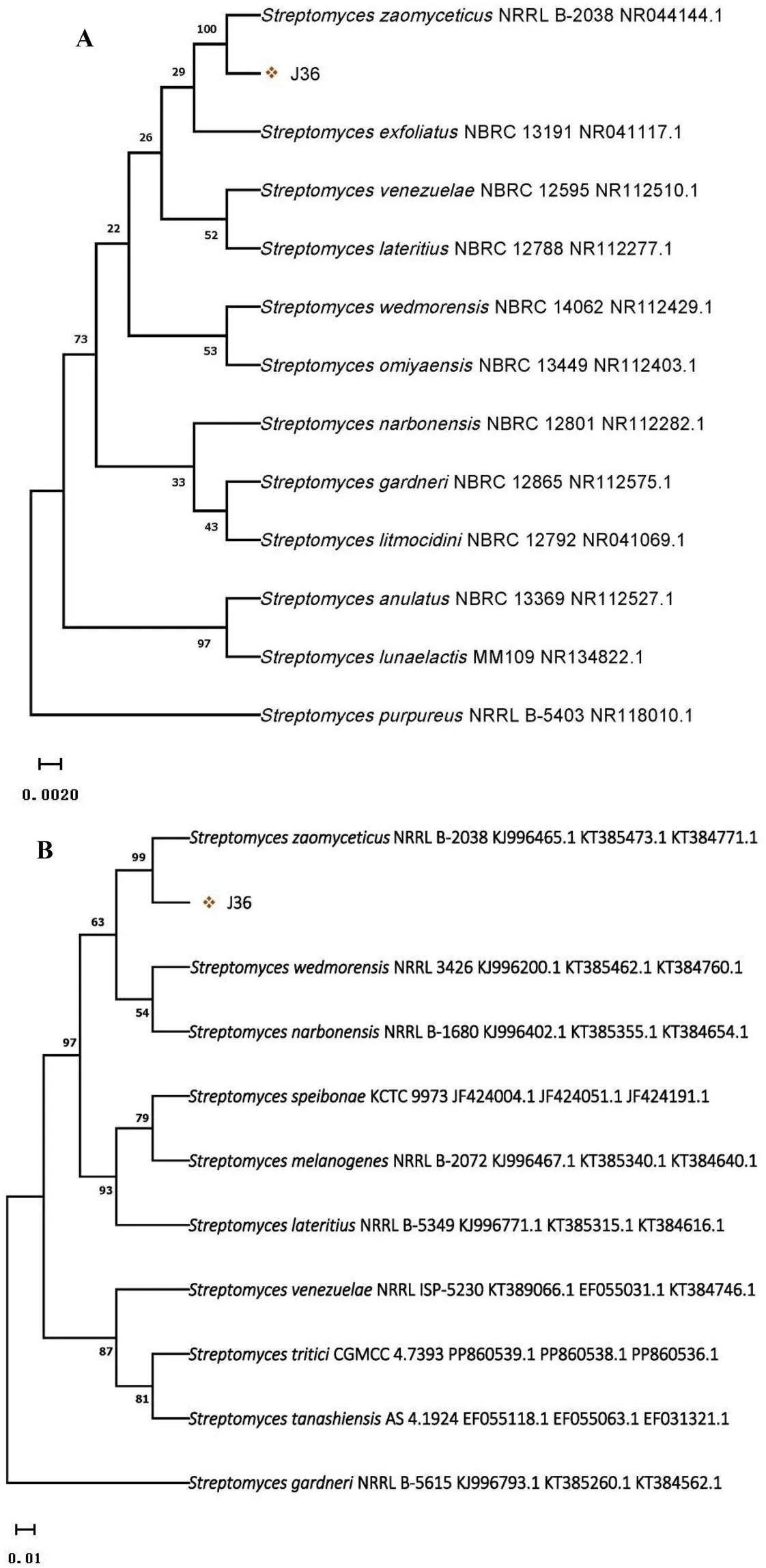
Phylogenetic trees of strain J36. **(A)** Phylogenetic tree of strain J36 based on 16S rRNA gene sequences. **(B)** Phylogenetic tree of strain J36 based on *rpoB*, *recA*, and *atpD* gene sequences.

The 16S rRNA gene sequence of strain J36 was compared against the EzBioCloud and NCBI databases. The results showed that strain J36 exhibited the highest similarity (100%) to *Streptomyces zaomyceticus* NRRL B-2038, followed by closely related species such as *S. exfoliatus*. A phylogenetic tree based on the 16S rRNA gene sequences was constructed using the maximum-likelihood (ML) method (Figure 7A), in which strain J36 clustered with *S. zaomyceticus* within the same subclade with a bootstrap value of 100%. However, the resolution of the 16S rRNA gene was insufficient to definitively identify the strain at the species level. To further clarify the taxonomic position of strain J36, multilocus sequence analysis (MLSA) was performed using three housekeeping genes, rpoB, recA, and atpD. An ML phylogenetic tree was constructed based on the concatenated nucleotide sequences of these three genes (Figure 7B), which revealed that strain J36 formed a robust and stable monophyletic subclade with *S. zaomyceticus* NRRL B-2038, supported by a bootstrap value of 99%. Phylogenetic analyses based on 16S rRNA gene sequences and MLSA revealed that strain J36 formed a tight cluster with *S. zaomyceticus*, suggesting a close phylogenetic relationship.

### The overall genome-related indexes

To evaluate the taxonomic position of strain J36, we calculated the average nucleotide identity (ANI) and digital DNA–DNA hybridization (dDDH) values against closely related species, which were selected from phylogenetic trees inferred from 16S rRNA gene and three housekeeping gene sequences. The highest values were observed with *Streptomyces zaomyceticus* NBC-00415, reaching 90.91% (ANI) and 39.30% (dDDH), respectively (Table 4). Both values fell well below the established thresholds for species demarcation (95% for ANI and 70% for dDDH; Meier-Kolthoff et al., 2022; Riesco and Trujillo, 2024). These results thus collectively indicate that strain J36 represents a novel species within the genus *Streptomyces*.

**Table 4:** The ANI and dDDH values between strain J36 and its closely related species.

Strains	ANI (%)	dDDH (%)
*Streptomyces zaomyceticus* NBC-00415^T^	90.91	39.30
*Streptomyces venezuelae* NRRL B-65442^T^	89.66	39.40
*Streptomyces gardneri* ATCC 15439^T^	87.07	33.50
*Streptomyces tanashiensis* Kala^T^	87.64	34.90
*Streptomyces exfoliatus* NRRL B-2924^T^	88.17	36.10

### CAZymes prediction and anti-oomycete activity of strain J36

**Figure 8:**
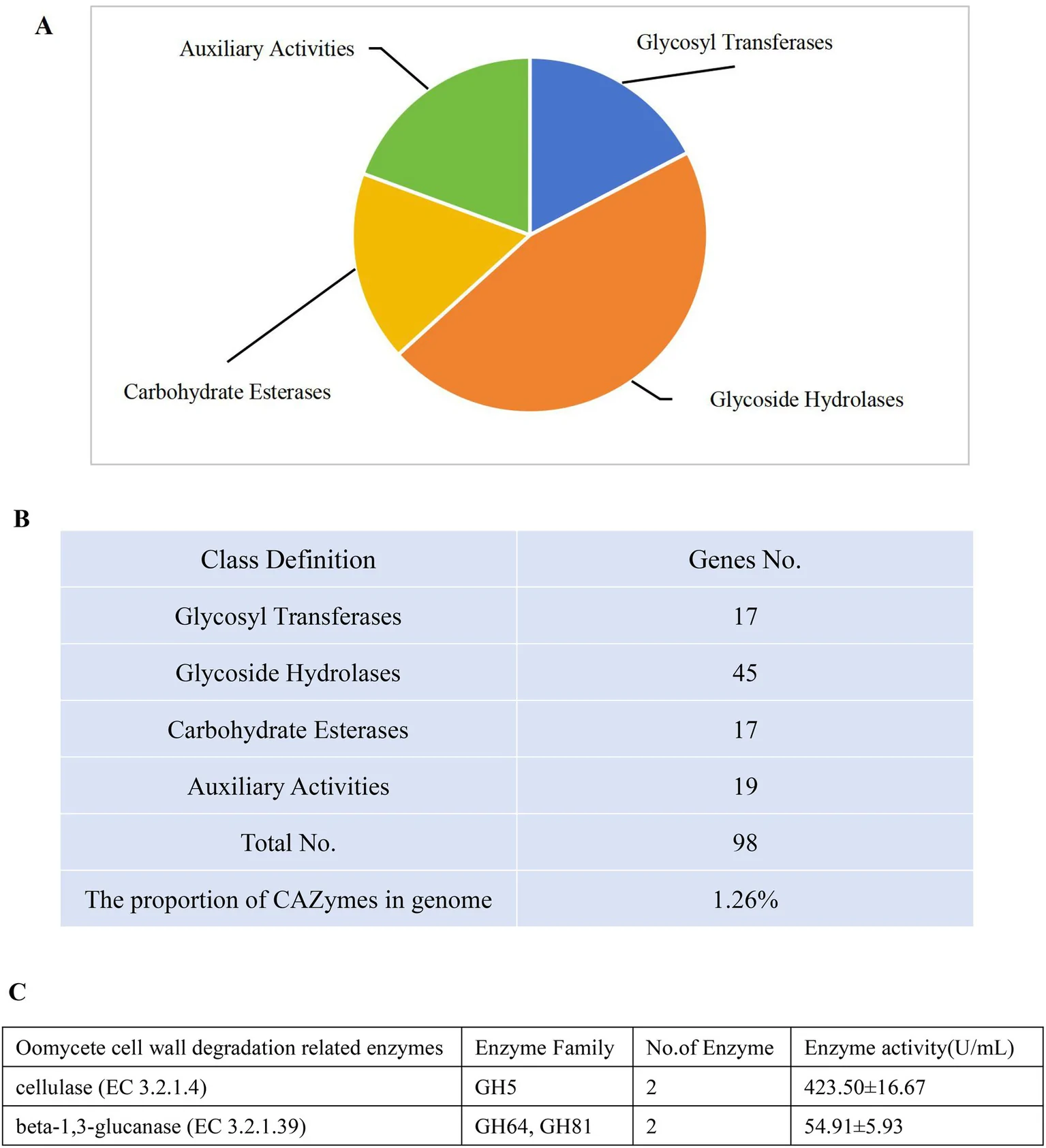
Prediction of CAZymes and determination of enzymatic activities of strain J36. **(A,B)** Statistical classification of CAZymes. **(C)** Cellulase and β-1,3-glucanase activities in the cell-free filtrate of strain J36 (*n* = 3).

To predict enzyme genes related to oomycete cell wall degradation, the CAZymes (carbohydrate-active enzymes) of strain J36 were annotated (Figure 8A). The results showed that the genome of strain J36 encoded 98 CAZymes, including 19 auxiliary activities (AAs), 17 carbohydrate esterases (CEs), 45 glycoside hydrolases (GHs), and 17 glycosyl transferases (GTs), with CAZymes accounting for 1.26% of the genome (Figure 8B). Additionally, two cellulases (EC 3.2.1.4) were identified in the GH5 family, and two β-1,3-glucanases (EC 3.2.1.39) were found in the GH64 and GH81 families. Cellulases and β-1,3-glucanases are often reported to degrade the cell walls of plant pathogenic oomycetes (Wilfred et al., 2025). To determine whether these enzymes are present in the cell-free filtrate of strain J36, the activities of cellulase and β-1,3-glucanase in the filtrate were determined to be 423.50 ± 16.67 U/mL and 54.91 ± 5.93 U/mL, respectively (Figure 8C), suggesting that these two enzymes may participate in the antagonistic activity of strain J36 against *P. cactorum*, although the filtrate contains a complex mixture of metabolites that may also be involved.

### Prediction of biosynthetic gene clusters (BGCs) in the genome of strain J36

The antiSMASH v8.0.4 analysis predicted 37 biosynthetic gene clusters for secondary metabolites within the J36 genome (Table 5). Among these, 10 BGCs were identified as being associated with polyketide synthase (PKS) and non-ribosomal peptide synthetase (NRPS) pathways, accounting for 27.03% of the total. Additional BGC types include three terpenes, three RiPPs, one melanin, one butyrolactone, one NI-siderophore, and one ectoine cluster. Several gene clusters showed high similarity to those responsible for known antimicrobial compounds, including venezuelin, cypemycin, *ε*-poly-L-lysine, and alnumycin, indicating the potential of strain J36 to produce bioactive antibiotics. Notably, 17 of the 37 predicted BGCs exhibited no significant similarity to any known clusters deposited in the MIBiG database, suggesting that strain J36 may harbor the genetic capacity to produce novel secondary metabolites with unknown biological activities. This genetic novelty, combined with the potent anti-oomycete activity of strain J36, highlights its potential as a promising source of bioactive natural products.

**Table 5:** Secondary metabolite clusters in strain J36 predicted by antiSMASH.

Region	Type	From	To	Most similar known cluster	Similarity confidence
1	Terpene-precursor	351,607	372,749	–	–
2	Lanthipeptide-class-iv	402,340	425,084	Venezuelin	High
3	NRPS, NAPAA	723,655	803,645	*–*	–
4	T2PKS, NRPS, T1PKS	896,290	1,069,728	Spore pigmen	High
5	Azole-containing-RiPP, terpene	1,149,507	1,192,287	*–*	–
6	NRP-metallophore, NRPS	1,214,336	1,307,369	Peucechelin	Medium
7	Melanin	1,444,188	1,454,580	Melanin	Low
8	Lanthipeptide-class-iii	1,616,212	1,638,956	SapB	High
9	RiPP-like	1,661,471	1,672,337	*–*	–
10	Terpene	1,725,301	1,751,922	Hopene	High
11	T2PKS, azole-containing-RiPP	1,888,863	1,961,408	Alnumycin	Medium
12	NRPS, T1PKS	1,991,027	2,053,740	Formicamycin	Low
13	Butyrolactone	2,146,892	2,157,839	Coelimycin	Low
14	RiPP-like	2,258,806	2,270,218	14-hydroxyisochainin	Low
15	NRPS	2,363,973	2,437,288	Pyrroloformamide	Medium
16	NRPS	2,452,163	2,510,848	–	–
17	NI-siderophore	2,545,076	2,578,204	Kinamycin	Low
18	NI-siderophore	2,613,587	2,644,891	–	–
19	Azole-containing-RiPP	2,940,316	2,973,660	–	–
20	Butyrolactone	2,977,871	3,018,980	A-factor	High
21	Terpene-precursor	3,202,939	3,223,994	–	–
22	Melanin	3,491,719	3,502,132	–	–
23	Linaridin	3,522,630	3,545,231	Cypemycin	High
24	NRPS-like	4,077,180	4,120,881	Lankamycin	Low
25	NI-siderophore	5,672,782	5,702,569	Desferrioxamin B	High
26	CDPS	6,314,043	6,334,771	–	–
27	NAPAA	7,671,128	7,710,836	ε-Poly-L-lysine	High
28	Lanthipeptide-class-ii, terpene	7,712,060	7,745,095	–	–
29	Terpene	8,036,735	8,058,933	Geosmin	High
30	Ectoine	8,101,616	8,112,032	Ectoine	High
31	NRPS, T1PKS, terpene-precursor	8,212,761	8,271,035	–	–
32	Hydrogen-cyanide	8,318,906	8,331,905	–	–
33	Lanthipeptide-class-iii	8,376,548	8,399,247	–	–
34	CDPS	8,449,153	8,469,911	–	–
35	Terpene	8,525,496	8,546,386	–	–
36	NRPS-like, T1PKS	8,612,807	8,678,682	–	–
37	Terpene	8,691,683	8,719,292	Isorenieratene	High

## Discussion

*Phytophthora cactorum* is a destructive oomycete pathogen that causes root rot disease in *P. notoginseng*, seriously threatening the sustainable development of the *P. notoginseng* industry (Ma et al., 2026). More than 75% of cultivated *P. notoginseng* varieties are susceptible to *P. cactorum*, and the disease often leads to root decay, wilting, and plant death, with an incidence rate exceeding 80% under rainy and high-humidity conditions, resulting in enormous economic losses. The use of antagonistic microorganisms for biocontrol of *P. notoginseng* root rot has gained increasing attention due to its environmentally friendly nature, low risk of resistance development, and ability to maintain rhizosphere microecological balance (Yu et al., 2022). To improve the efficacy of biological control and overcome the limitations of conventional management strategies, screening for broad-spectrum, highly efficient, and stable novel antagonistic microorganisms as biocontrol agents is of critical importance (Wang et al., 2024). Unique environments, such as the rhizosphere of continuously cropped *P. notoginseng* fields and soils from high-altitude cultivation areas, often harbor diverse functional microorganisms that serve as valuable resources for the discovery of novel biocontrol agents.

In this study, 72 actinobacterial strains with distinct morphological characteristics were isolated from soils collected from a *P. notoginseng* cultivation base in Wenshan City. Among them, 31 strains exhibited antagonistic activity against *P. cactorum*, with strain J36 demonstrating the strongest anti-oomycete activity. To contextualize the biocontrol potential of strain J36, we compared its performance with previously reported *Streptomyces* strains active against *Phytophthora* species. In terms of inhibitory activity, documented antagonistic *Streptomyces* exhibit varying inhibition rates against *Phytophthora* pathogens. For instance, *Streptomyces lateritius* ZY37 achieved a 76.91% inhibition rate against *P. sojae* after 7 days of dual-culture incubation (Liu H. et al., 2025; Liu Y. P. et al., 2025), and *Streptomyces* sp. PR69 exhibited inhibition rates 73% against *P. capsici* under the same incubation period (López Reyes et al., 2024). Notably, strain J36 displayed a mean inhibition rate of 63.44% against *P. cactorum* after only 5 days of dual-culture incubation, a level comparable to those reported for strains cultured for 7 days.

On the basis of phenotypic characteristics and phylogenetic analyses of 16S rRNA, rpoB, recA, and atpD genes, strain J36 was shown to be closely related to *Streptomyces zaomyceticus*. The 16S rRNA gene sequence of strain J36 shared 100% similarity with that of *S. zaomyceticus*, yet due to its conserved nature, it cannot provide sufficient resolution for species discrimination. Therefore, we employed MLSA using the three protein-coding genes, which exhibited higher divergence. The resulting phylogenetic trees placed strain J36 and *S. zaomyceticus* NRRL B-2038 in a well-supported clade (bootstrap 99%), confirming their close affinity. Nevertheless, genome-based ANI and dDDH comparisons between strain J36 and *S. zaomyceticus* yielded values below the species cutoff thresholds, which unequivocally indicate that strain J36 should be recognized as a novel species within the genus *Streptomyces*.

Genome sequencing of strain J36 revealed a linear chromosome of 8,755,751 bp with a GC content of 72.43%, encoding 7,779 protein-coding genes. The GC content is consistent with the typical genomic features of actinobacteria, which are known for their high GC contents (typically 70–74%). CAZymes annotation identified 98 carbohydrate-active enzymes in the genome, accounting for 1.26% of the total genes, including two cellulases (GH5) and two β-1,3-glucanases (GH64 and GH81). These enzyme families are known to be involved in the degradation of oomycete cell walls, which are mainly composed of cellulose and β-1,3-glucans (Wilfred et al., 2025). Consistent with the genomic predictions, enzymatic assays demonstrated that the cell-free filtrate of strain J36 possessed cellulase and β-1,3-glucanase activities of 423.50 ± 16.67 U/mL and 54.91 ± 5.93 U/mL, respectively. These results suggest that the anti-oomycete activity of strain J36 is at least partially attributed to the production of cellulase and β-1,3-glucanase, which may act synergistically to degrade the cell wall of *P. cactorum*. In addition to the potent inhibitory activity, the genomic features of strain J36 further support its biocontrol potential. In terms of secondary metabolite biosynthesis, whole-genome sequencing of *S. luteireticuli* ASG80 identified a total of 40 biosynthetic gene clusters (BGCs) encompassing various known antimicrobial compound types (Xu et al., 2024). Through antiSMASH prediction, the genome of strain J36 was found to contain 37 BGCs, including 3 NRPS and 7 PKS gene clusters, which are frequently associated with the production of known antimicrobial compounds in *Streptomyces* (Hwang et al., 2014). Compared with ASG80, the number of BGCs in J36 remains at a relatively high level. Moreover, 17 of the 37 BGCs showed no significant similarity to any known clusters in the MIBiG database, suggesting that this novel species may possess unique secondary metabolite resources, which could represent an additional important source of its potent antagonistic activity.

Collectively, our findings demonstrate that strain J36 is a potent producer of cell wall-degrading enzymes and exhibits significant anti-oomycete activity against *P. cactorum*. The combination of taxonomic identification, genomic analysis, and enzymatic characterization provides robust evidence supporting the potential of J36 as a promising biocontrol agent for the management of *P. notoginseng* root rot. Several *Streptomyces* strains have been reported to exhibit biocontrol activity against *Phytophthora* diseases (Xu et al., 2024; Wang et al., 2025). To the best of our knowledge, this is the first report on a systematic genomic and enzymatic activity analysis of a strain belonging to this novel *Streptomyces* species for the biocontrol of *Phytophthora* root rot of *P. notoginseng*. Future studies will focus on optimizing fermentation conditions to enhance enzyme production, evaluating its efficacy under field conditions, and elucidating the molecular mechanisms underlying its antagonistic activity.

## Conclusion

In this study, 72 actinobacterial strains were isolated from the rhizosphere soil of *P. notoginseng* and screened for their antagonistic activity against *P. cactorum*. Among them, strain J36, isolated from the rhizosphere of *P. notoginseng*, exhibited strong anti-oomycete activity. Based on morphological, physiological, and biochemical characteristics, as well as phylogenetic analyses of the 16S rRNA, rpoB, recA, and atpD genes, strain J36 was found to be closely related to *Streptomyces zaomyceticus*. However, the calculated ANI and dDDH values supported its recognition as a novel species of the genus *Streptomyces*. Whole-genome sequencing and assembly revealed a chromosome of 8,755,751 bp with a GC content of 72.43%, encoding 7,779 protein-coding genes. In addition, genome mining identified a broad array of biosynthetic gene clusters, including 17 putatively novel ones. CAZymes annotation revealed that the genome harbors multiple glycoside hydrolase families associated with oomycete cell wall degradation, and enzymatic assays further confirmed the activities of cellulase and β-1,3-glucanase in the cell-free filtrate. Collectively, these results suggest that cellulase and β-1,3-glucanase produced by strain J36 may be involved in its anti-oomycete activity, providing a preliminary basis for its potential as a biocontrol candidate.

## Data Availability

The data presented in this study have been deposited to the NCBI GenBank (https://www.ncbi.nlm.nih.gov/genbank). The GenBank accession numbers for the 16S rRNA, rpoB, recA, and atpD genes of strain J36 are PZ120913, PZ148826, PZ148827, and PZ148828, respectively. This Whole Genome project has been deposited under the GenBank accession JBYEQP000000000. The version described in this paper is version JBYEQP010000000.

## References

[ref1] Besemer J. Borodovsky M. (2005). GeneMark: web software for gene finding in prokaryotes, eukaryotes and viruses. Nucleic Acids Res. 33, W451–W454. doi: 10.1093/nar/gki487, 15980510 PMC1160247

[ref2] Bhatti A. A. Haq S. Bhat R. A. (2017). Actinomycetes benefaction role in soil and plant health. Microb. Pathog. 111, 458–467. doi: 10.1016/j.micpath.2017.09.036, 28923606

[ref3] Bolger A. M. Lohse M. Usadel B. (2014). Trimmomatic: a flexible trimmer for Illumina sequence data. Bioinformatics 30, 2114–2120. doi: 10.1093/bioinformatics/btu170, 24695404 PMC4103590

[ref4] Cantarel B. L. Coutinho P. M. Rancurel C. Bernard T. Lombard V. Henrissat B. (2009). The carbohydrate-active EnZymes database (CAZy): an expert resource for Glycogenomics. Nucleic Acids Res. 37, D233–D238. doi: 10.1093/nar/gkn663, 18838391 PMC2686590

[ref5] Delcher A. L. Bratke K. A. Powers E. C. Salzberg S. L. (2007). Identifying bacterial genes and endosymbiont DNA with glimmer. Bioinformatics 23, 673–679. doi: 10.1093/bioinformatics/btm009, 17237039 PMC2387122

[ref6] Guo R. J. Liu X. Z. Li S. D. Miao Z. Q. (2009). In vitro inhibition of fungal root-rot pathogens of *Panax notoginseng* by rhizobacteria. Plant Pathol. J. 25, 70–76. doi: 10.5423/ppj.2009.25.1.070

[ref7] Hu S. Li K. Zhang Y. Wang Y. Fu L. Xiao Y. . (2022). New insights into the threshold values of multi-locus sequence analysis, average nucleotide identity and digital DNA–DNA hybridization in delineating *Streptomyces* species. Front. Microbiol. 13:910277. doi: 10.3389/fmicb.2022.910277, 35711787 PMC9195134

[ref8] Hwang K. Kim U. H. Charusanti P. Palsson B. Ø. Lee S. Y. (2014). Systems biology and biotechnology of *Streptomyces* species for the production of secondary metabolites. Biotechnol. Adv. 32, 255–268. doi: 10.1016/j.biotechadv.2013.10.008, 24189093

[ref9] Hyatt D. Chen G. L. LoCascio P. F. Land M. L. Larimer F. W. Hauser L. J. (2010). Prodigal: prokaryotic gene recognition and translation initiation site identification. BMC Bioinformat. 11:119. doi: 10.1186/1471-2105-11-119, 20211023 PMC2848648

[ref10] Li Y. B. Liu Y. X. Zhang Z. P. Li J. Q. Zhu S. S. Yang M. . (2022). Application of plant survival-promoting and pathogen-suppressing *Trichoderma* species for crop biofertilization and biocontrol of root rot in *Panax notoginseng*. J. Plant Pathol. 104, 1361–1369. doi: 10.1007/s42161-022-01166-3

[ref11] Liang D. Andersen C. B. Vetukuri R. R. Dou D. Grenville L. J. (2020). Horizontal gene transfer and tandem duplication shape the unique CAZyme complement of the mycoparasitic oomycetes *Pythium oligandrum* and *Pythium periplocum*. Front. Microbiol. 11:581698. doi: 10.3389/fmicb.2020.581698 33329445 PMC7720654

[ref12] Liu H. Hao C. Zhang Z. Xu Y. Jiao X. Zhang H. . (2025). Biocontrol potential of *Streptomyces lateritius* ZY37 against *Phytophthora* root rot in soybean and optimization of fermentation conditions. J. Appl. Microbiol. 136, 1–10. doi: 10.1093/JAMBIO/LXAF175, 40693993

[ref13] Liu Y. P. Hu Y. Li Y. Tian G. X. Luo L. F. Zhao S. H. . (2025). *Mortierella alpina* bioinoculant potentiates native microbiota for soil borne disease suppression in *Panax notoginseng* cultivation. Pestic. Biochem. Physiol. 214:106615. doi: 10.1016/J.PESTBP.2025.106615, 40915803

[ref14] López Reyes P. K. De la Torre Zavala S. Cortés González M. M. Galán Wong L. J. Avilés Arnaut H. (2024). Biological control of *Streptomyces* sp. PR69 against *Phytophthora capsici* and its growth-promoting effects on plants. Horticulturae 10, 1365–1365. doi: 10.3390/HORTICULTURAE10121365

[ref15] Ma S. Chen Z. J. Xiong G. Cheng M. Wang Y. Sun Y. . (2026). Comparative response of root-associated soil bacterial communities to *Phytophthora* blight in healthy versus diseased *Panax notoginseng* plants. Sci. Rep. 16:25005. doi: 10.1038/S41598-026-56433-5 42225795 PMC13462700

[ref16] Meier-Kolthoff J. P. Sardà Carbasse J. Peinado-Olarte R. L. Göker M. (2022). TYGS and LPSN: a database tandem for fast and reliable genome-based classification and nomenclature of prokaryotes. Nucleic Acid Res. 50, D801–D807. doi: 10.1093/NAR/GKAB902, 34634793 PMC8728197

[ref17] Naughton L. M. Romano S. O’Gara F. Dobson A. D. W. (2017). Identification of secondary metabolite gene clusters in the *Pseudovibrio* genus reveals encouraging biosynthetic potential toward the production of novel bioactive compounds. Front. Microbiol. 8:1494. doi: 10.3389/fmicb.2017.01494, 28868049 PMC5563371

[ref18] Qi D. Liu Q. Zou L. Zhang M. Li K. Zhao Y. . (2024). Taxonomic identification and antagonistic activity of *Streptomyces luomodiensis* sp. nov. against phytopathogenic fungi. Front. Microbiol. 15:1402653. doi: 10.3389/FMICB.2024.1402653, 38860218 PMC11163044

[ref19] Qi D. Zou L. Zhou D. Zhang M. Wei Y. Zhang L. . (2021). Identification and antifungal mechanism of a novel actinobacterium *Streptomyces huiliensis* sp. nov. against *fusarium oxysporum* f. sp. cubense tropical race 4 of banana. Front. Microbiol. 12:722661. doi: 10.3389/FMICB.2021.722661, 34803941 PMC8600237

[ref20] Riesco R. Trujillo M. E. (2024). Update on the proposed minimal standards for the use of genome data for the taxonomy of prokaryotes. Int. J. Syst. Evol. Microbiol. 74:006300. doi: 10.1099/IJSEM.0.006300, 38512750 PMC10963913

[ref21] Rong X. Huang Y. (2010). Taxonomic evaluation of the *Streptomyces griseus* clade using multilocus sequence analysis and DNA-DNA hybridization, with proposal to combine 29 species and three subspecies as 11 genomic species. Int. J. Syst. Evol. Microbiol. 60, 696–703. doi: 10.1099/ijs.0.012419-0, 19656940

[ref22] Shirling E. B. Gottlieb D. (1966). Methods for characterization of *Streptomyces* species. Int. J. Syst. Evol. Microbiol. 16, 313–340. doi: 10.1099/00207713-16-3-313, 27077644

[ref23] Tatusova T. DiCuccio M. Badretdin A. Chetvernin V. Nawrocki E. P. Zaslavsky L. . (2016). NCBI prokaryotic genome annotation pipeline. Nucleic Acids Res. 44, 6614–6624. doi: 10.1093/nar/gkw569, 27342282 PMC5001611

[ref24] Wang Y. Jia F. Song R. Chen C. Pu T. Xu Y. . (2025). Characterization and genome analysis of marine *Streptomyces albidoflavus* S20 for the biological control of tobacco black shank. Biol. Control 211:105908. doi: 10.1016/J.BIOCONTROL.2025.105908

[ref25] Wang Z. Wang S. Yang H. (2024). Understanding the pathogenesis, biocontrol mechanisms, and factors influencing biocontrol effectiveness for soil-borne diseases in panax plants. Microorganisms 12:2278. doi: 10.3390/MICROORGANISMS12112278, 39597667 PMC11596276

[ref26] Wang C. X. Zhao X. L. Wu K. Liang C. Y. Liu J. Yang H. . (2023). Isolation and characterization of *Bacillus velezensis* strain B19 for biocontrol of *Panax notoginseng* root rot. Biol. Control 185:105311. doi: 10.1016/J.BIOCONTROL.2023.105311

[ref27] Weisburg W. G. Barns S. M. Pelletier D. A. Lane D. J. (1991). 16S ribosomal DNA amplification for phylogenetic study. J. Bacteriol. 173, 697–703. doi: 10.1128/jb.173.2.697-703.1991, 1987160 PMC207061

[ref28] Wick R. R. Judd L. M. Gorrie C. L. Holt K. E. (2017). Unicycler: resolving bacterial genome assemblies from short and long sequencing reads. PLoS Comput. Biol. 13:e1005595. doi: 10.1371/journal.pcbi.1005595, 28594827 PMC5481147

[ref29] Wilfred M. A. Chen S. Zhao Y. Lu J. Chen Y. Zhou D. . (2025). Regulation of *Trichoderma harzianum* gene expression and protein production in submerged cultures with inactivated oomycete mycelium. Microbiol. Res. 303:128378. doi: 10.1016/J.MICRES.2025.128378 41223685

[ref30] Xu G. Wu W. Zhu L. Liang Y. Liang M. Tan S. . (2024). Whole genome sequencing and biocontrol potential of *Streptomyces luteireticuli* ASG80 against *Phytophthora* diseases. Microorganisms 12:2255. doi: 10.3390/MICROORGANISMS12112255, 39597644 PMC11596116

[ref31] Yu S. F. Wang C. L. Hu Y. F. Wen Y. C. Sun Z. B. (2022). Biocontrol of three severe diseases in soybean. Agriculture 12:1391. doi: 10.3390/agriculture12091391

